# A single center cross-sectional study examining the prevalence of inpatient postpartum anxiety and post-traumatic stress symptoms using the State-Trait Anxiety Inventory and Post-Traumatic Stress Disorder Checklist for DSM-5

**DOI:** 10.18103/mra.2026.0452

**Published:** 2026-08

**Authors:** Claudia Lucia Sotillo, Guillermina Michel, Sana Shah, Margaret Smith, Sarah Ciechanowicz, Z Bekemeyer, Aleesha Jasmin Jethwa, Brendan Carvalho, Nan Guo, Michael Joshua Ostacher, Debra Lee Kaysen, Danielle Mari Panelli, Pervez Sultan10

**Affiliations:** 1 Department of Anesthesiology, Perioperative and Pain Medicine, Stanford University School of Medicine, California, USA; 2 University of California, Irvine School of Medicine, California, USA; 3 California Pacific Medical Center (CPMC), San Francisco, California, USA; 4 Department of Anaesthesia and Perioperative Medicine, University College London Hospital, London, England, United Kingdom; 5 Emory University, Atlanta, Georgia; 6 Department of Psychiatry and Behavioral Sciences. Stanford University School of Medicine, California, USA; 7 Department of Obstetrics & Gynecology, Maternal Fetal Medicine. Stanford University School of Medicine, California, USA

**Keywords:** PTSD, anxiety, Postpartum, Prevalence, STAI, PCL-5

## Abstract

**Background:**

Mental health conditions are now the leading cause of maternal mortality in the United States, prompting calls for universal postpartum anxiety and post-traumatic stress disorder screening. However, standardized guidelines for screening implementation remain deficient. This cross-sectional study evaluated the prevalence of inpatient postpartum anxiety and post-traumatic stress disorder while exploring associated risk factors.

**Objectives:**

Over a three-week period, we prospectively assessed adult postpartum patients following all delivery modes (vaginal and cesarean, live and stillbirth) at a single academic institution in the US. Screening was performed within 36 hours of delivery using the State-Trait Anxiety Inventory (score cut off ≥80) and post-traumatic stress disorder checklist for DSM-5 (score cut off ≥28). Data collection included demographic, medical, anesthesia, obstetric and neonatal variables, with assessments conducted in-person or via telephone for any spoken language.

**Study design:**

Cross-sectional study

**Results:**

Among 275 eligible patients, 263 (95.6%) completed the screening measures. The prevalence of positive postpartum anxiety and post-traumatic stress disorder screening was 12.5% and 4.9% respectively, with 3.8% of patients screening positive for both conditions. Worse State-Trait Anxiety Inventory scores were associated with ASA ≥3, pre-term birth and neonatal Apgar scores at 5 minutes <7. Worse post-traumatic stress disorder scores were associated with ASA ≥3, primiparity, spontaneous vaginal delivery, pre-eclampsia, pre-term birth, blood transfusion, Apgar score <7 and neonatal intensive care unit admission. Factors after adjustment associated with positive anxiety screening were a psychiatric history or medication use. No factors after adjustment were associated with positive PTSD screening.

**Conclusions:**

Positive screening for inpatient postpartum anxiety was two and a half times more prevalent than for post-traumatic stress disorder. Positive anxiety screening was independently associated with psychiatric history or medication use. Targeted screening for post-traumatic stress disorder may be more appropriate, particularly among patients with pre-existing psychiatric conditions, complicated pregnancies or neonatal morbidity. Future research should focus on determining optimal screening parameters and timing, and intervention guidelines to better support high-risk obstetric populations.

## Introduction

The United States (US) faces a concerning maternal health crisis, with current maternal mortality rates estimated at 22.3 deaths per 100,000 live births.^1^ Mental health conditions, particularly postpartum depression, post-traumatic stress disorder (PTSD), and anxiety, are now the leading cause of maternal death in the US, accounting for 28% of fatalities within one year following delivery.^2^ These conditions frequently co-occur, posing significant risks that not only affect the mother, but can also affect maternal-infant bonding, breastfeeding efficacy and long-term infant cognitive and behavioral development.^3-9^ In response, the US Preventative Services Task Force (USPSTF) now recommends universal depression and anxiety screening for perinatal patients.^10^ However, standardized guidelines for the timing of anxiety and PTSD screening, methodology and subsequent therapeutic interventions are lacking.^11-13^

Historical understanding of anxiety and PTSD is largely derived from studies of combat trauma in predominantly white, English-speaking male populations.^12,14,15^ Recent research has begun to examine these conditions specifically in postpartum patients, revealing that 13-40% of mothers experience postpartum anxiety disorders, with 34% describing their childbirth as traumatic.^16-20^ Community rates of postpartum PTSD range from 3-4%, rising to 16-19% in high-risk populations.^5,8^ However, existing studies show considerable methodological variation, including use of heterogeneous measures, variable timing of screening and selection bias in terms of English language used to screen patients, which often limits their generalizability.^21-25^

Importantly, anxiety and PTSD are distinct conditions, which can co-occur.^9^ Anxiety is more prevalent and may therefore potentially be suited to universal screening, whereas PTSD is comparatively rare and may be more efficiently detected through risk-stratified screening.^10,12^ The inpatient postpartum admission in the US is an accessible point for healthcare contact, providing a small window of several days to identify at-risk patients before discharge, however there is limited contemporary information on screening within this population.^26^ Given the lack of guidelines supporting timing of screening for anxiety and PTSD, snap-shot audits can play a valuable role in determining inpatient prevalence rates of positive screening and help determine clinical feasibility of introducing routine screening of all patients who deliver in this setting.^27^

The primary aim of this quality improvement initiative was to determine the prevalence of positive anxiety screening in all postpartum patients during inpatient hospitalization, regardless of delivery mode. Secondary aims were to determine the incidence of postpartum PTSD screening and explore differences in State-Trait Anxiety Inventory (STAI) and PTSD Checklist for DSM-5 (PCL-5) scores between clinical groups (American Society of Anesthesiologists (ASA) physical status, parity, delivery mode, pre-eclampsia, pre-term birth, hemorrhage, blood transfusion, Apgar score and neonatal intensive care unit (NICU) admission) and identify demographic, medical, anesthesia, obstetric and neonatal variables associated with positive screening for anxiety and PTSD.

## Methods

This quality improvement project adopted a cross-sectional study design, which received exemption from the institutional review board. The findings from this initiative would be used to determine the feasibility of adopting routine clinical screening for anxiety and PTSD at our institution for all inpatient postpartum patients. This decision would be based on positive screening rates and the anticipated workload for mental health response staff. These results are reported in accordance with the Strengthening the Reporting of Observational Studies in Epidemiology (STROBE) guidelines.^28^

All adult postpartum patients at a US academic center were eligible for screening over a three-week period between June and July 2024. Inclusion criteria encompassed all adult postpartum patients regardless of English proficiency (all languages were included), age, delivery type, or pregnancy outcome (including viable and non-viable fetuses). Patients were excluded if they were <18 years old, did not undergo a vaginal or cesarean delivery (e.g. cervical cerclage or dilatation and evacuation procedures) or if they refused to answer surveys.

Trained investigators conducted screening between 12 and 36 hours postpartum using the standardized scripts shown in Table 1, for both in-person and telephone encounters to minimize response bias.

Patients requiring maternal intensive care admission were approached after they were well enough to be transferred to the postpartum ward. Those discharged before 36 hours postpartum (such as following uncomplicated vaginal delivery) were contacted via telephone and offered email-based screening. Spanish translations and interpreter services were provided when necessary. Cyracom interpreting services (Tucson, Arizona) loaded onto electronic tablets were used for patients that did not speak or understand English as per hospital protocol.

Two validated screening instruments were employed: (i) the State-Trait Anxiety Inventory (STAI Form Y): A 40-item assessment previously validated as the optimal screening measure for postpartum anxiety according to COnsensus-based Standards for the selection of health Measurement INstruments (COSMIN) guidelines.^21,29^ A cut-off value of ≥80 was used as previously described.^30^ (ii) the PTSD Checklist for DSM-5 (PCL-5): A 20-item diagnostic tool aligned with DSM-5 criteria and validated in a large multicenter prospective study.^31^ A cut-off value of ≥28 was used as previously used in the postpartum population.^31^ Investigators also collected comprehensive baseline data from the electronic health records and patient report including: demographic (age, height, weight, body mass index (BMI), primary language, education level, ethnicity, race, household income within the last 12 months, marital status, employment status); medical (previous psychiatric history, family psychiatric history, psychiatric medications); anesthesia (ASA physical status); obstetric (gestational age, gravidity, parity, delivery mode, comorbidities, pre-term birth, fetal anomalies, postpartum hemorrhage, blood transfusion); and neonatal (Apgar scores at 5 minutes, NICU admission) variables.

All data were directly entered into REDCap (Research Electronic Data Capture; https://www.project-redcap.org), a secure web-based platform accessed via institutional tablets. Patients screening positive for postpartum anxiety or PTSD were discussed with the anesthesiology attending on call and their obstetric care team and then referred for appropriate social work and psychiatric services as deemed necessary. Follow-up care protocols were established for all patients who screened positive.

The primary outcome for this quality improvement initiative was the prevalence of positive anxiety screening (STAI ≥ 80). Secondary outcomes were the prevalence of positive PTSD screening (PCL-5 ≥28) and to explore differences in STAI and PCL-5 scores between the following patient groups: a) ASA physical status; b) multiparous vs primiparous; c) cesarean vs vaginal delivery; d) pre-eclampsia vs no pre-eclampsia diagnosis; e) pre-term vs no pre-term birth (<37 vs ≥37 weeks); f) postpartum hemorrhage ≥1.5 L vs blood loss <1.5 L; g) blood transfusion vs no blood transfusion; h) Apgar score at 5 minutes <7 vs ≥7; and i) NICU admission vs no NICU admission. Finally, we sought to identify variables (demographic, medical, anesthesia, obstetric and neonatal) associated with positive screening for anxiety and PTSD adjusted for potential confounders. We hypothesized that STAI and PCL-5 scores would be higher in patients with the following characteristics; demographic (underrepresented minority groups), medical (pre-existing mental health diagnosis), obstetric (hemorrhage, blood transfusion) and neonatal (low Apgar scores at 5 minutes and NICU admission).

Based on published literature and the known acuity of our patient population, we hypothesized that 20% and 15% of our inpatient postpartum cohort would screen positive for anxiety and PTSD, respectively.^21,31^ Based on institutional delivery data, we anticipated the screening a convenience sample of approximately 276 patients over a 3-week period (186 vaginal deliveries and 90 cesarean deliveries, including 51 intrapartum and 39 scheduled procedures), to provide adequate numbers for prevalence data.

Mean (SD) or Median (IQR [range]) values were calculated for normally and non-normally distributed continuous variables, respectively. Categorical data were presented as count (percentage). One-way Analysis of variance (ANOVA) was used to compare continuous variables and Chi-square test or Fisher’s Exact test was used to compare categorical variables. STAI and PCL-5 scores were compared between patient groups using ANOVA. The associations between the demographic, medical, anesthesia, obstetric and neonatal variables with positive screening for anxiety were explored in logistic regressions and all confounders were fit in the multivariable logistic regression. Multi-collinearity was checked by Variance Inflation Factor (VIF) and VIF>5 were considered high multicollinearity. Statistical significance was set at P<0.05, and all analyses were performed using Stata Version 14.0 (StataCorp., College Station, TX).

## Results

During the three-week data collection period, 275 patients delivered. A summary of the number of completed surveys during the study period is provided in Figure 1. The median data collection time for STAI and PCL-5 was 25.6 hours postpartum, and response rates were 96.4% and 95.6%, respectively.

Table 2 summarizes the demographics and Table 3 summarizes the key medical, anesthesia, obstetric and neonatal variables for the patients that delivered.

The prevalence of positive anxiety screening in this delivery cohort was 12.5% (33/263). The prevalence of positive PTSD screening was 4.9% (13/263). The prevalence of co-existing anxiety and PTSD was 3.8% (10/263). The prevalence for positive PTSD screening without concurrent anxiety was 1.1% (3/263).

Table 4 summarizes the differences in STAI and PCL-5 scores between different patient groups. STAI scores were significantly higher in patients with the following characteristics: higher ASA physical status, pre-term birth and lower Apgar score at 5 minutes. PCL-5 scores were significantly higher in patients with the following characteristics: higher ASA physical status, primiparous, spontaneous vaginal delivery, pre-eclampsia, pre-term birth, blood transfusion, lower Apgar score at 5 minutes, NICU admission.

After adjustment, a psychiatric history or medication use (all drug types pooled) was associated with a STAI score ≥80 (Table 5).

No factors were significantly associated with a PCL-5 score ≥28 after adjustment (Table 6).

## Discussion

### PRINCIPAL FINDINGS

In our inpatient cohort screened within 36 hours of delivery, positive screening for postpartum anxiety and PTSD occurred in 12.5% and 4.9%, respectively. Prior psychiatric history or medication use was an independent risk factor for positive anxiety screening. Although the univariate analyses for both conditions showed significant associations with ASA physical status, pre-term birth and lower Apgar scores, these were not shown to be independently associated in the multivariable logistic regression model. These findings suggest that early postpartum anxiety and PTSD symptoms may be associated with a combination of non-modifiable factors (prior psychiatric history, ASA physical status, pre-term birth and lower Apgar scores) and unmeasured, potentially modifiable factors related to the acute stress of delivery (e.g. adequacy of pain control, quality of communication from the care team, availability of peripartum psychological support).^32-34^ The absence of independent predictors of positive PTSD screening after adjustment likely reflects the low number of positive screens and the study being inadequately powered for this outcome, highlighting the need for larger prospective studies to determine optimal targeting strategies for inpatient PTSD screening.

These findings are relevant as recent USPSTF recommendations call for national postpartum anxiety screening, however fail to provide guidance regarding optimal timing of screening.^10^ Our study sought to determine if inpatient screening within 36 hours of delivery would have a significant prevalence that justifies the potential workforce burden to screen all patients. Our 12.5% prevalence for positive anxiety screening aligns with previously reported rates of 13-40% in the literature.^16,26,35-37^ Our institutional rate of positive postnatal depression screening, using the Edinburgh Postnatal Depression Scale, is 12%. This suggests that universal anxiety screening during the inpatient period may also be justified but must be weighed against the increased staffing workload involved in education and implementation of universal screening and increased survey burden for postpartum patients. Screening patients with previous psychiatric diagnoses and medication requirements may be beneficial, as this emerged as a strong independent risk factor for positive anxiety screening. The lower prevalence of positive PTSD screening (4.9%) and the 1.1% prevalence of positive screening for PTSD without co-existing anxiety indicates that a more targeted approach may be more appropriate for PTSD screening. Such an approach could focus on high-risk groups such as patients who experience delivery related complications including pre-eclampsia, pre-term birth, fetal anomalies, blood transfusion or delivery of a neonate requiring NICU admission.

The prevalence of inpatient postpartum anxiety in our study cohort (12.5%) is lower than other prevalence studies. A study in Qatar using the Depression, Anxiety, and Stress Scale reported a similar postpartum anxiety prevalence of 13%.^35^ However studies utilizing the STAI, our chosen measure, reported rates ranging from 17% in an American randomized controlled trial of post-discharge care models to 23% at one week postpartum and up to 40% in other cohorts.^36,26,37^ Regarding postpartum PTSD, our prevalence rate (4.9%) is consistent with other studies. An Australian study using the Post-traumatic Diagnostic Scale, while controlling for pre-existing conditions, found rates of 1.2% at 4-6 weeks postpartum, increasing to 3.1% at both 12 and 24 weeks postpartum.^38^ In contrast, a French study utilizing the Peritraumatic Distress Inventory (PDI) and Perinatal Posttraumatic Stress Disorder Questionnaire (PPQ) reported notably higher rates, with 11.7% at 2 months and 10.5% at 6 months postpartum.^39^ Our lower reported prevalence rates may reflect screening in the immediate postpartum period, which may increase after patients have time to process events.

### CLINICAL IMPLICATIONS

After adjusting for confounding factors, we found that race and ethnicity did not significantly impact anxiety screening outcomes in our cohort. Our comparatively low number of positive PTSD screens provides valuable information for decision-making regarding implementation of universal screening and for powering future studies. The univariate analysis findings identify specific patient populations who might benefit most from early targeted interventions, particularly those with peripartum complications such as pre-eclampsia, pre-term birth, fetal anomalies or with lower Apgar scores. These findings can help institutions optimize their screening protocols and allocation of resources in order to identify patients who may benefit most from early targeted intervention.

Prior studies have demonstrated a higher proportion of anxiety in postpartum working women with higher education or premature delivery, however these findings were not reproducible in our population after adjusting for other confounding factors. ^35,40,39^

The assessment and diagnosis of postpartum anxiety and PTSD present significant methodological challenges across existing research. Prior prospective and database studies have shown considerable variability in their approaches, including differences in study definitions, screening measures, cut-off scores, patient populations, and assessment timing (i.e. postpartum week/month) and location (i.e. inpatient vs outpatient). Furthermore, historical data suggest that positive screening results often fail to correlate with formal ICD-9 or ICD-10 diagnoses, potentially leading to underestimation of conditions such as anxiety and PTSD.^41^ To address these methodological concerns, our study employed validated screening measures with literature-supported cut-off scores. We selected the STAI based on a prior COSMIN criteria validation study, which identified it as the optimal clinical screening measure for postpartum anxiety.^21^ Similarly, we utilized the PCL-5 for its validated effectiveness in identifying postpartum PTSD.^31^

### RESEARCH IMPLICATIONS

The optimal cut-off values for both STAI and PCL-5 that correlate with clinically significant morbidity have yet to be established.^31,42^ Although developing postpartum-specific screening tools could enhance sensitivity and specificity, such measures would require rigorous validation before clinical implementation.^31^ Furthermore, the management of patients who screen positive for anxiety and PTSD in the inpatient setting presents logistical challenges.^43^ At our institution, for example, social work referral typically precedes psychiatric evaluation due to resource constraints, a common scenario given that many healthcare facilities lack adequate mental health services to manage the approximate 13% of patients who screen positive in the early postpartum period. The trajectory and time to resolution of anxiety and PTSD symptoms also remains poorly understood, yet this information is crucial for determining optimal screening timing, referral and treatment protocols. Future prospective studies comparing inpatient and outpatient symptom trajectories are needed to better understand delayed presentation patterns and inform screening strategies across the postpartum continuum.

## Strengths and limitations

Our inclusion of nearly all patients who delivered at our institution over a three-week period, regardless of language spoken, addresses limitations of previous prospective studies, which have often been restricted to predominantly white, middle-class, educated, married, and English-speaking patients.^44,45^ The study population reflected broad diversity across primary language spoken, ethnicity, race, and educational level, with a notably high representation of Hispanic patients (36.5%), and 28% of the included cohort exclusively spoke Spanish. However, as a single center study conducted in a US academic setting, our findings may still have limited generalizability to rural or private healthcare settings across other institutions. The timing of PTSD screening during the immediate postpartum period may not have been optimal, as screening is typically more appropriate 1-2 weeks after an acute stress event, and formal PTSD diagnosis requires at least one-month post-event. We likely underestimated the true prevalence of PTSD, as patients who experienced severe traumatic events such as stillbirth were more likely to decline or not complete the screening measures, and the relatively low number of positive PTSD screens in our cohort suggests that a larger sample size would be beneficial for future studies. Finally, while the screening tools used are validated in this population, they may not have captured the full spectrum of mental health concerns in this population, such as depression.

## Conclusion

In conclusion, inpatient postpartum anxiety was two and half times more prevalent than PTSD. We found that a history of psychiatric history or medication use was a strong independent risk factor for positive anxiety screening. Future studies are needed to develop postpartum anxiety and PTSD screening and referral guidelines, and to design early targeted interventions for patients at risk of these disorders.

## Figures and Tables

**Figure 1.**
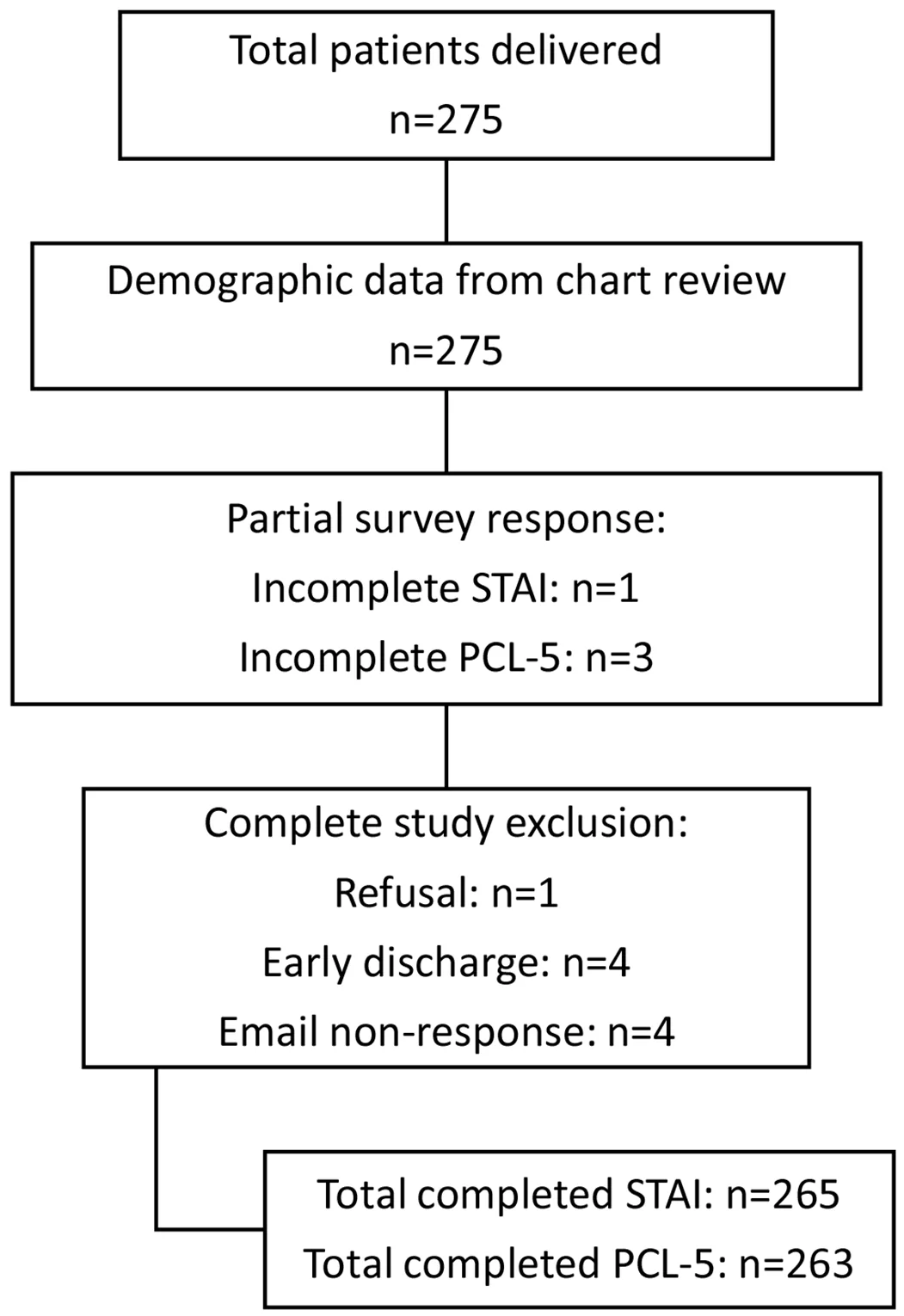
Flow diagram of survey completion PCL-5= PTSD Checklist for DSM-5; STAI=State-Trait Anxiety Inventory.

**Table 1:** Standardized bilingual recruitment script used to invite postpartum patients to complete mental health screening surveys

Language	Script
English	Hello, My name is ________. I am part of the Obstetric/Obstetric Anesthesiology team here at Stanford. We are working on a new initiative to improve mental health for mothers. As a part of this initiative, everyone who is delivering is being asked important questions to improve patient care. We would like you to spare 10 minutes of your time to help us with this wellness initiative. We hope to use this information to improve care for all postpartum patients. → Yes: Thank you for being so generous with your time! I will facilitate your completion of these brief surveys with this ipad. → No: Would it be best if I come at a different time?
Spanish	Buenos dias, Me llamo _______. Soy parte del equipo Obstétrico/de Anestesia Obstétrica aquí en Stanford. Estamos trabajando en una nueva iniciativa para mejorar la salud mental para todas las madres. Como parte de esta iniciativa, todas las mujeres que están dando a luz están recibiendo varias preguntas importantes para mejorar el cuidado de todas las pacientes. Le pedimos 10 minutos de su tiempo para ayudarnos con esta iniciativa. Esperamos usar la información colectada para mejorar el cuidado de todas las mujeres postparto. → Si: iGracias por prestarnos un poco de su tiempo! Voy a ayudarla a completar estas encuestas con este ipad. → No: ¿Sería mejor si regreso más tarde?

The script was used for in-person and telephone encounters and included prompts for patients who agreed or declined participation.

**Table 2:** Demographic characteristics of the delivery cohort who completed the State-Trait Anxiety Inventory and PTSD Checklist DSM-5 surveys

	Delivery cohort n=275
Age (yr)	31.9±5.9
Height (cm)	162.2±7.7
Antenatal Weight (kg)	78.3±14.2
Antenatal BMI (kg/m^2^)	29.8±5.2
Primary language	
English	200 (72.7%)
Spanish	64 (23.3%)
Other	11 (4.0%)
Education level	
High school or below	61 (22.2%)
Some college, no degree	21 (7.6%)
College degree	66 (24.0%)
Postgraduate degree	103 (37.5%)
Missing	24 (8.7%)
Ethnicity	
Hispanic	101 (36.7%)
Non-Hispanic	174 (63.3%)
Race	
Asian	94 (34.2%)
Black	3 (1.1%)
White	72 (26.2%)
Other or mixed race	61 (22.2%)
Unknown	45 (13.4%)
Household income within last 12 months	
< $25,000	40 (14.6%)
$25,000 - $50,000	23 (8.4%)
$50,000 - $100,000	17 (6.2%)
$100,000 - $150,000	16 (5.8%)
> 150,000	118 (42.9%)
Prefer not to say	61 (22.2%)
Marital status	
Single	35 (12.7%)
Married	190 (69.1%)
Living with partner	29 (10.6%)
Prefer not to say	21 (7.6%)
Employment status	
Not working	44 (16.0%)
Working full time	114 (41.5%)
Working part time	17 (6.2%)
Student	6 (2.2%)
Other	8 (2.9%)
Missing	86 (31.3%)

BMI=body mass index; values presented as number (percentage)

**Table 3:** Medical, anesthesia, obstetric and neonatal characteristics of the delivery cohort who completed the State-Trait Anxiety Inventory and PTSD Checklist DSM-5 surveys

	Delivery cohort n=275
Medical / anesthesia variables	
Previous psychiatric history	
Major depressive disorder	20 (7.3%)
Anxiety	33 (12.0%)
Bipolar	1 (0.4%)
PTSD	4 (1.5%)
*Other psychiatric history	13 (4.7%)
Family psychiatric history	11 (4.0%)
Psychiatric medications	
SSRI	8 (2.9%)
SNRI	1 (0.4%)
**Other psychiatric medications	4 (1.5%)
ASA physical status	
2	243 (88.4%)
3	29 (10.6%)
Unknown	3 (1.1%)
Obstetric variables	
Gestational age	37.9±3.0
Gravidity	
1	114 (41.6%)
2	65 (23.7%)
More than 2	95 (34.7%)
Parity	
0	141 (51.5%)
1	80 (29.2%)
More than 1	53 (19.3%)
Delivery mode	
Vaginal delivery	187 (68.0%)
Cesarean delivery	86 (31.3%)
Unknown	2 (0.7%)
Obstetric comorbidities	
GDM	45 (16.4%)
Multiple gestation	5 (1.8%)
Gestational hypertension/Pre-eclampsia	40 (14.6%)
Chorioamnionitis	5 (1.8%)
Pre-term birth (<37 weeks)	30 (10.9%)
Fetal anomalies	18 (6.6%)
PPH < 1.5L	257 (93.4)
PPH ≥1.5L	13 (4.7%)
Blood transfusion	10 (3.6%)
***Other obstetric comorbidities	139 (50.6%)
Neonatal variables	
Apgar score at 5 minutes <7	12 (4.4%)
Apgar score at 5 minutes ≥7	259 (94.2)
NICU admission	20 (7.3%)

ASA=American Society of Anesthesiologists; GDM=gestational diabetes; L=liter; NICU=neonatal intensive care unit; PPH=postpartum hemorrhage; PTSD=post-traumatic stress disorder; SNRI=serotonin-norepinephrine reuptake inhibitor; SSRI=selective serotonin reuptake inhibitor; values presented as mean ± standard deviation and number (percentage).

* Other psychiatric history includes attention-deficit, eating, obsessive-compulsive, adjustment, substance use, psychotic, personality, and other specified psychiatric disorders.

** Other psychiatric medications include atypical antidepressants, mood stabilizers, antipsychotics, anxiolytics, and stimulant medications.

*** Other obstetric comorbidities include obstetric and fetal complications, placental abnormalities, infertility or pregnancies conceived through in vitro fertilization, endocrine and metabolic disorders, hematologic and coagulation disorders, cardiopulmonary conditions, infections, genetic carrier status, and gastrointestinal, neurologic, psychiatric, autoimmune, and musculoskeletal conditions.

**Table 4:** Differences in State-Trait Anxiety Inventory and PTSD Checklist DSM-5 scores between patient cohorts

	STAI score	p-value	PCL-5	p-value
Medical / anesthesia variables				
ASA physical status:		**0.049**		**<0.001**
Grade 2 vs	60 (16)		6 (9)	
Grade 3	61 (20)		9 (14)	
Obstetric variables				
Parity:		0.058		**0.004**
Multiparous vs	60 (17)		4 (10)	
primiparous birth	60 (15)		6 (8)	
Delivery mode:		0.095		**0.001**
Cesarean delivery vs	62 (18)		6 (8)	
SVD	59 (15)		8 (11)	
Pre-eclampsia:		0.706		**0.008**
Pre-eclampsia vs	61 (17)		8 (12)	
no pre-eclampsia	60 (16)		6 (9)	
Pre-term birth:		**<0.001**		**<0.001**
Pre-term birth vs	67 (24)		13 (15)	
no pre-term birth	59 (15)		6 (8)	
Hemorrhage:		0.268		0.616
PPH ≥1.5L vs	57 (13)		8 (8)	
Blood loss <1.5L	60 (16)		6 (9)	
Blood transfusion:		0.063		**<0.001**
Transfusion vs	71 (23)		14 (20)	
no transfusion	59 (16)		6 (9)	
Neonatal variables				
Apgar score at 5 minutes:		**0.001**		**0.004**
<7 vs	78 (28)		15 (16)	
≥7	59 (15)		6 (9)	
NICU admission:		0.072		**<0.001**
NICU admission vs	68 (21)		11 (16)	
no NICU admission	59 (15)		6 (9)	

NICU=neonatal intensive care unit; PCL-5=PTSD Checklist for DSM-5; PPH=postpartum hemorrhage; STAI=State-Trait Anxiety Inventory; SVD=spontaneous vaginal delivery. Values are presented as median (interquartile range). P-values are from group comparisons; values in **bold** indicate statistical significance (P < 0.05).

**Table 5:** Factors associated with a State-Trait Anxiety Inventory score ≥80

	Unadjusted OR (95%	p	Adjusted OR (95%	p
Demographic variables				
Age (yr)	1.04 (0.98, 1.12)	0.194	0.96 (0.85, 1.09)	0.573
Primary Language				
Non-English	**Ref**		Ref	
English	**4.38 (1.29, 14.81)**	**0.018**	2.87 (0.39, 21.30)	0.303
Ethnicity				
Non-Hispanic	**Ref**		Ref	
Hispanic	**0.34 (0.14, 0.87)**	**0.024**	0.49 (0.06, 4.07)	0.513
Race				
White	Ref		Ref	
Non-white	0.67 (0.30, 1.52)	0.341	0.80 (0.30, 2.08)	0.641
Household income within last 12 months				
≤$150,000	Ref		Ref	
>150,000	1.19 (0.54, 2.63)	0.668	0.49 (0.15, 1.54)	0.222
Marital status				
Single	Ref		Ref	
Married or living with partner	5.43 (0.76, 41.13)	0.102	2.62 (0.25, 27.36)	0.422
Medical / anesthesia variables				
Psychiatric history or medication	**5.16 (2.36, 11.25)**	**<0.001**	**3.59 (1.30, 9.87)**	**0.013**
ASA physical status				
2	Ref		Ref	
3	0.85 (0.24, 2.98)	0.795	0.53 (0.06, 4.48)	0.560
Obstetric Variables				
Gestational age				
37-42 weeks	Ref		Ref	
<32 weeks	2.23 (0.44, 11.30)	0.332	0.45 (0.02, 11.41)	0.626
32-36 weeks	2.84 (0.85, 9.55)	0.091	0.39 (0.04, 3.79)	0.416
Primiparous vs multiparous	0.87 (0.42, 1.81)	0.709	1.20 (0.45, 3.16)	0.715
Delivery mode				
Vaginal delivery	Ref		Ref	
Cesarean delivery	1.44 (0.68, 3.05)	0.345	0.89 (0.32, 2.48)	0.825
*Obstetric comorbidities	1.19 (0.55, 2.59)	0.659	1.07 (0.39, 2.96)	0.889
Fetal anomalies	1.99 (0.53, 7.55)	0.311	1.60 (0.23, 11.41)	0.637
Blood transfusion	3.73 (0.89, 15.72)	0.072	6.01 (0.69, 52.68)	0.105
Neonatal variables				
Apgar at 5 minutes <7	**3.21 (1.05, 9.8)**	**0.040**	2.78 (0.38, 20.58)	0.316

OR=odds ratio; aOR=adjusted odds ratio; CI=confidence interval; ASA=American Society of Anesthesiologists; STAI=State-Trait Anxiety Inventory. Reference categories are indicated as “Ref”. Values in **bold** indicate statistical significance (P < 0.05).

* Obstetric comorbidities include gestational diabetes, multiple gestation, gestational hypertension/pre-eclampsia, and chorioamnionitis.

**Table 6:** Factors associated with PTSD Checklist for DSM-5 score ≥28

	OR (95% CI)	p-value	aOR (95% CI)	p-value
Demographic variables				
Age (yr)	1.04 (0.94, 1.15)	0.148	1.05 (0.83, 1.32)	0.689
Primary language				
Non-English	Ref		Ref	
English	2.18 (0.47, 10.09)	0.318	0.25 (0.01, 4.61)	0.349
Ethnicity				
Non-Hispanic	Ref		Ref	
Hispanic	0.51 (0.14, 1.89)	0.311	-	
Race				
White	Ref		Ref	
Non-white	0.64 (0.17, 2.34)	0.499	0.36 (0.04, 2.94)	0.339
Household income within last 12 months				
≤$150,000	Ref		Ref	
>150,000	0.53 (0.15, 1.94)	0.338	0.34 (0.04, 2.70)	0.307
Medical / anesthesia variables				
Psychiatric history or medication	**8.66 (2.69, 27.85)**	**<0.001**	6.06 (0.93, 39.60)	0.060
ASA physical status				
2	Ref		Ref	
3	2.79 (0.72, 10.83)	0.139	0.28 (0.01, 12.72)	0.514
Obstetric variables				
Gestational age				
37-42 weeks	**Ref**		Ref	
<32 weeks	**8.21 (1.47, 45.98)**	**0.017**	1.95 (0.02, 189.74)	0.775
32-36 weeks	**7.19 (1.69, 30.59)**	**0.008**	1.40 (0.11, 17.77)	0.111
Primiparous vs multiparous	0.40 (0.12, 1.33)	0.136	0.73 (0.11, 4.94)	0.749
Delivery method				
Vaginal delivery	Ref		Ref	
Cesarean delivery	2.59 (0.84, 7.97)	0.097	1.00 (0.16, 6.21)	0.997
*Obstetric comorbidities	2.08 (0.68, 6.40)	0.202	3.88 (0.56, 26.88)	0.169
Fetal anomalies	**6.52 (1.57, 27.10)**	**0.010**	5.43 (0.28, 104.27)	0.262
Blood transfusion	**6.31 (1.17, 33.99)**	**0.032**	3.41 (0.15, 76.03)	0.438
Neonatal variables				
Apgar at 5 minutes < 7	**5.01 (1.24, 20.30)**	**0.024**	12.01 (0.31, 461.29)	0.182

OR=odds ratio; aOR=adjusted odds ratio; CI=confidence interval; ASA=American Society of Anesthesiologists; PCL-5=PTSD Checklist for DSM-5. Reference categories are indicated as “Ref”. Values in **bold** indicate statistical significance (P < 0.05).

* Obstetric comorbidities include gestational diabetes, multiple gestation, gestational hypertension/pre-eclampsia, and chorioamnionitis.
